# Clinical characteristics and outcomes of phase I cancer patients with CCNE1 amplification: MD Anderson experiences

**DOI:** 10.1038/s41598-022-12669-5

**Published:** 2022-05-24

**Authors:** Shuyang Yao, Funda Meric-Bernstam, David Hong, Filip Janku, Aung Naing, Sarina Anne Piha-Paul, Apostolia Maria Tsimberidou, Daniel Karp, Vivek Subbiah, Timothy Anthony Yap, Jordi Rodon Ahnert, Shubham Pant, Ecaterina E Ileana Dumbrava, Chetna Wathoo, Erick Campbell, Lihou Yu, Yuko Yamamura, Siqing Fu

**Affiliations:** 1 Department of Thoracic Surgery, Xuanwu Hospital Capital Medical University, Beijing, China; 2 Department of Investigational Cancer Therapeutics, Unit 0455, The University of Texas MD Anderson Cancer Center, 1515 Holcombe Boulevard, Houston, TX 77030 USA; 3 Institute of Personalized Cancer Therapy, The University of Texas MD Anderson Cancer Center, Houston, TX USA

**Keywords:** Cancer, Molecular biology, Oncology

## Abstract

Cyclin E is frequently encoded by CCNE1 gene amplification in various malignancies. We reviewed the medical records of patients with solid tumors displaying CCNE1 amplification to determine the effect of this amplification for future therapeutic development. We reviewed the medical records of patients with advanced solid tumors harboring CCNE1 amplification who were seen at the phase I clinic between September 1, 2012, and December 31, 2019. Among 79 patients with solid tumors harboring CCNE1 amplification, 56 (71%) received phase 1 clinical trial therapy, 39 (49%) had 3 or more concurrent genomic aberrances, and 52 (66%) had a concurrent TP53 mutation. The median overall survival (OS) after patients’ initial phase I visit was 8.9 months and after their initial metastasis diagnosis was 41.4 months. We identified four factors associated with poor risk: age < 45 years, body mass index ≥ 25 kg/m^2^, presence of the TP53 mutation, and elevated LDH > upper limit of normal. In patients treated with gene aberration-related therapy, anti-angiogenic therapy led to significantly longer OS after their initial phase I trial therapy than those who did not: 26 months versus 7.4 months, respectively (P = 0.04). This study provided preliminary evidence that CCNE1 amplification was associated with frequent TP53 mutation and aggressive clinical outcomes. Survival benefit was observed in patients who received antiangiogenic therapy and gene aberration-related treatment, supporting the future development of a personalized approach to combine gene aberration-related therapy with antiangiogenesis for the treatment of advanced malignancies harboring CCNE1 amplification.

## Introduction

Cancer is characterized by uncontrolled cell proliferation associated with abnormal cell cycle activity and genomic instability^1^. Cell cycle progression is orchestrated by the orderly expression of cyclins (regulatory subunits), which sequentially activate cyclin-dependent kinases (CDKs)^2^, governing the cell division machinery. Cyclin E (E1 and E2) is the most abundant between G1 and S-phase of the cell cycle. Once Cyclin E binds to Cdk2 to form the unique configuration that is required for the transition from the G1 to S phase of the cell cycle, it initiates DNA duplication^3^. Cyclin E demonstrates high oncogenic potential, which can induce tumor formation both in vivo and in vitro^4–6^. The *CCNE1* gene, which encodes for cyclin E, is frequently amplified in various histologic subtypes and sites of primary disease, ranging from 0.4 to 40.4%^7^ per The Cancer Genome Atlas (TCGA) PanCan 2018 dataset^3,8–15^. CCNE1 amplification was identified more frequently in patients with primary platinum resistant and refractory epithelial ovarian cancer, which was associated with poor survival, evolving as a potential predictive factor of cytotoxic chemotherapy resistance, as well as a promising target for novel therapy^16,17^.

In the present study, we reviewed the demographic characteristics and clinical outcomes of patients with CCNE1 amplification in advanced solid tumors who were referred to the Clinical Center for Targeted Therapy (CCTT) at the University of Texas MD Anderson Cancer Center in an effort to better understand the characteristics of patients with *CCNE1* amplified malignancies, as well as to explore therapeutics opportunities for these patients.

## Patients and methods

### Patient selection

Our study included patients with advanced solid tumors harboring *CCNE1* amplification who were seen in the Clinical Center for Targeted Therapy (CCTT) at The University of Texas MD Anderson Cancer Center between September 1, 2012, and December 31, 2019. Patient demographics, medical histories, Eastern Cooperative Oncology Group (ECOG) performance status and laboratory results from their initial office visit, gene aberration results, and outcomes of treatments that were administered at the CCTT were obtained from patients’ electronic medical records. This study was conducted in accordance with the guidelines of the MD Anderson Institutional Review Board (IRB), and we were granted the ethical approval (RCR05-0270) for this study.

### CCNE1 amplification detection

Tumor tissues and/or circulating cell-free DNA (cfDNA) were analyzed for CCNE1 amplification on multiple genomic testing platforms, such as Oncomine Comprehensive Assay CMS400^18^, Foundation^one^ CDx^19^, and Guardant360^20^.

### Phase I trial treatment and evaluation

The decision of whether to enroll an eligible study patient in a phase I clinical trial depended on protocol availability and the discretion of the treating physician. Tumor responses were evaluated according to the Response Evaluation Criteria in Solid Tumors (RECIST) (version 1.1)^21^. All patients were followed until death or censored on August 21, 2020. Progression-free survival (PFS) was defined as the time from study entry to the date of first objective documentation of progressive disease, date of death, or censor date. Overall survival (OS) was defined as the time from the date of the first visit to the CCTT (OS-phase I), or as the date of the initial metastasis diagnosis (OS-metastasis) to the date of death or censor date, regardless of whether they received a phase I trial therapy. The phase 1 clinical trial therapy was considered to be a matched therapy if the patient received one or more agents targeting an actionable genetic aberration or protein downstream from it, such as HER2 antibodies for HER2 overexpression or amplification^22,23^ or a mitogen-activated protein kinase (MEK) inhibitor for a BRAF mutation^24^.

### Statistical analyses

Categorical data were summarized with use of frequencies. PFS and OS curves were plotted by using the Kaplan–Meier method and were compared by using log-rank tests. A Cox proportional hazard model was used to determine prognostic. All tests were two-sided and considered significant when *P* values were less than 0.05. Statistical analyses were performed with use of the SPSS software program (version 25; IBM Corporation).

## Results

### Patient characteristics

**Figure 1:**
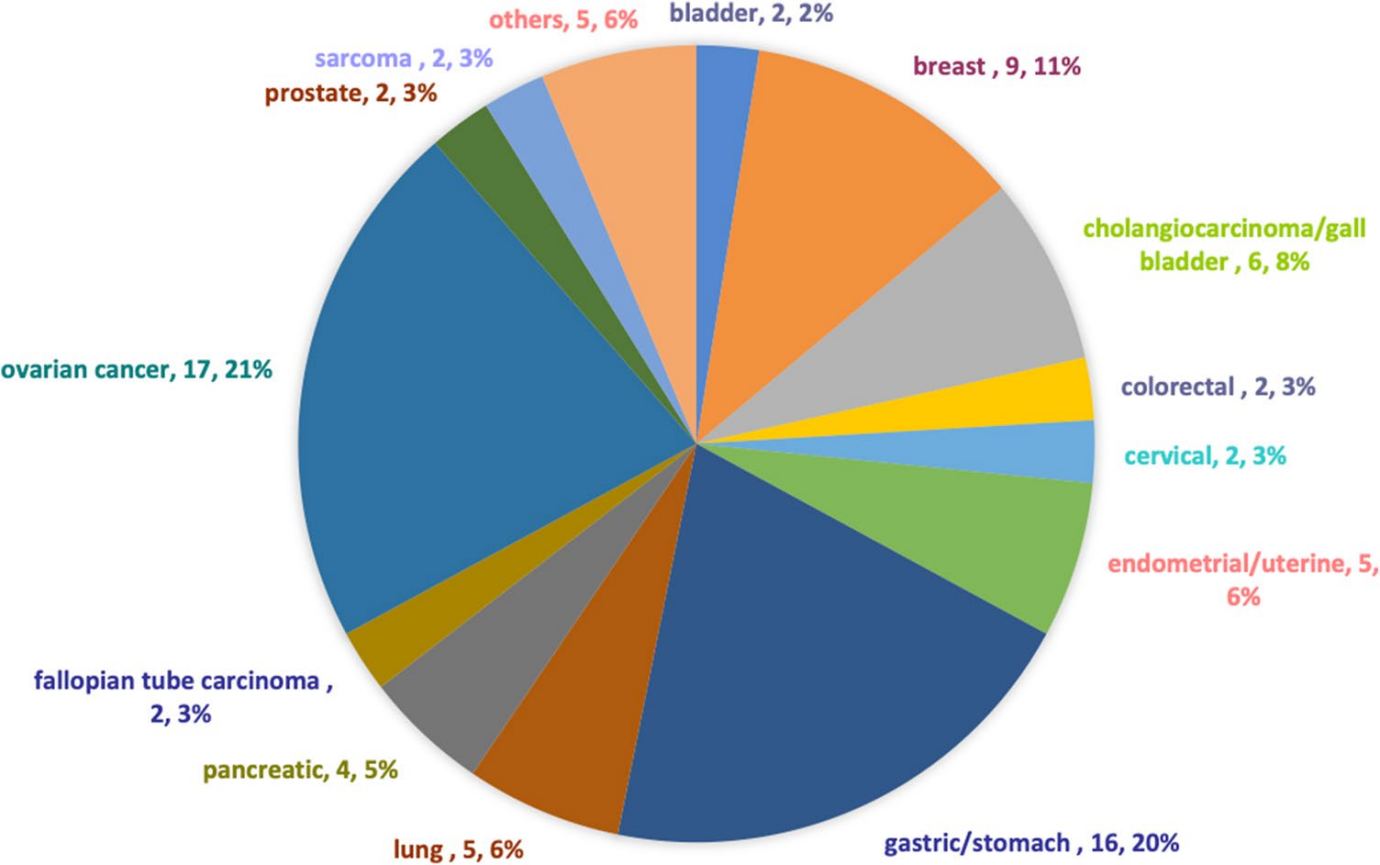
A pie chart demonstrates frequency of CCNE1 amplification per tumor type in patients who were seen at a designated phase I cancer trial service (n = 79).

Of 79 patients with recurrent or metastatic advanced solid tumors harboring CCNE1 amplification seen at the CCTT, approximately 71% of patients (n = 56) received phase 1 clinical trial therapy. Table 1 showed the baseline demographic data. The primary diagnoses included bladder (n = 2), breast (n = 9), cholangiocarcinoma/gall bladder (n = 6), colorectal (n = 2), cervical (n = 2), endometrial/uterine (n = 5), gastric (n = 16), lung (n = 5), pancreatic (n = 4), ovarian cancer(n = 19), prostate (n = 2), sarcoma (n = 2), and others (n = 5, primary peritoneal, adrenal gland, oropharynx, small intestine and appendix each), as shown in Fig. 1. In patients with adenocarcinoma as the leading pathologic type, approximately 90% had poorly differentiated or high-grade disease. Other pathologic types included transitional cell carcinoma, adrenocortical carcinoma, squamous carcinoma, clear cell carcinoma and urothelial carcinoma. Secondary primary tumors were identified in more than 20% of these patients (n = 18).

**Table 1:** Baseline demographic data.

Patient characteristics	Total N = 79 (%)
Median age, years (range)	60 (20–76)
**Sex**	
Female	46 (58.2)
Male	33 (41.8)
**Race**	
White	57 (72.2)
Black	11 (13.9)
Hispanic	2 (2.5)
Asian	5 (6.3)
Unknown	4 (5.1)
**Pathology**	
Adenocarcinoma	32 (40.5)
Neuroendocrine carcinoma	6 (7.6)
Serous carcinoma	19 (24.1)
Ductal carcinoma	8 (10.1)
Sarcoma	9 (11.4)
Others	5 (6.3)
**Differentiated**	
Moderately-differentiated	7 (8.9)
Poorly-differentiated	68 (86)
Unknown	4 (5.1)
**BMI**	
≥ 25	38 (48.1)
< 25	41 (51.9)
≥ 1 other primary cancer	18 (22.8)
History of VTE	17 (21.5)
ECOG PS ≤ 1	74 (93.7)
Elevated LDH	31 (39.2)
Low albumin	8 (10.1)
≥ 2 metastatic sites	62 (78.5%)
Prior surgery	50 (63.3)
Prior radiation	44 (55.7)
Prior immunology treatment	7 (8.9)
Prior systemic treatment (median number, range)	3 (1–9)

*N* number, *VTE* venous thromboembolism, *BMI* body mass index.

### Concurrent molecular aberrations

In these 79 patients with *CCNE1* amplified cancer, concurrent genetic aberrations were found in approximately 94% of these patients all patients (n = 74; 93%), and three or more concomitant genetic aberrations were found in 39 patients (49%): *TP53* (n = 52; 66%), *PIK3CA* (n = 19; 24%), *KRAS* (n = 14; 18%), *AKT1/2* (n = 9; 11%), *BRAF* (n = 9; 11%), *PTEN* (n = 9; 11%), *MYC* (n = 8; 10%), *MET* (n = 6; 8%), *BRCA1* (n = 5; 6%), and *EGFR* (n = 5; 6%).

### Phase I trial therapy

**Figure 2:**
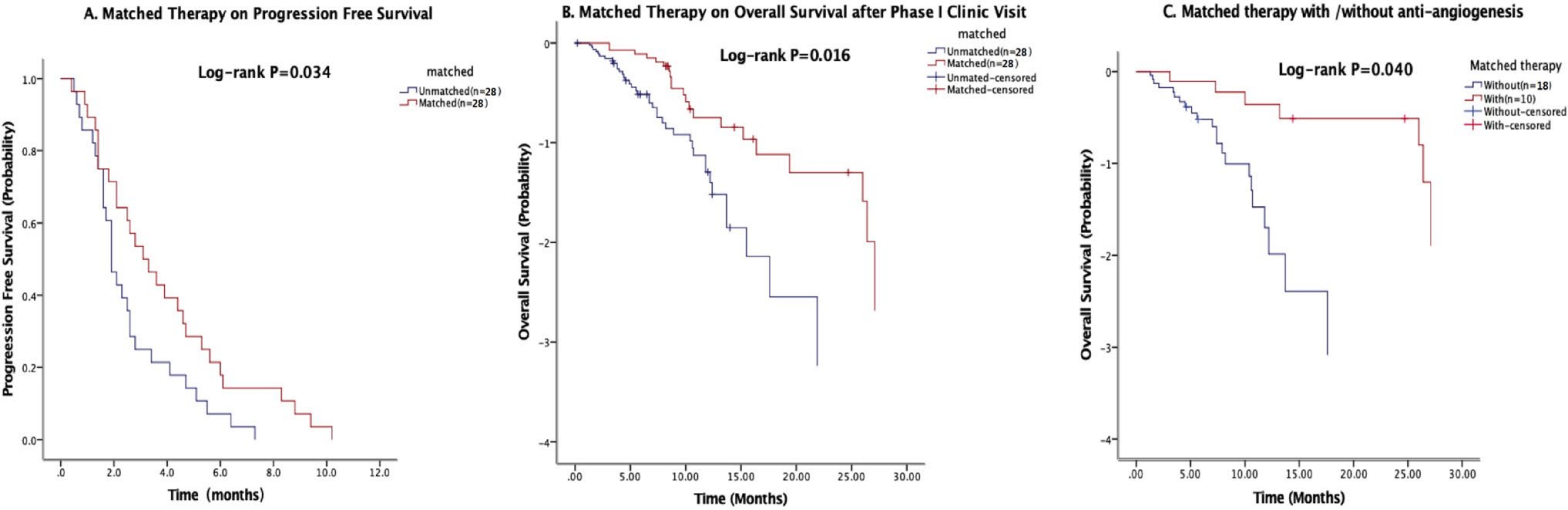
Kaplan–Meier curves estimate progression-free survivals in patients with CCNE1 amplification who received matched phase 1 trial therapy versus those who did not (**A**); Kaplan–Meier curves estimate overall survival after initial phase I trial clinic visit in patients with CCNE1 amplification who received matched phase 1 trial therapy versus those who did not (**B**); Kaplan–Meier curves estimate overall survival after initial phase I trial clinic visit in patients with CCNE1 amplification who received matched phase 1 trial therapy, stratified by anti-angiogenesis treatment (**C**).

A total of 56 patients received phase I trial treatments. The median PFS was 2.5 months (95% confidence interval [CI], 1.9–3.1 months). Among these patients, 28 received therapy matched to their concurrent genetic aberrations: c-MET pathway (n = 2), BRAF/MEK pathway (n = 6), PIK3CA pathway (n = 2), HER2 pathway (n = 9), WEE1 inhibition (n = 4), anti-CD30 antibody (n = 1), CDK4-6 inhibition (n = 1), EGFR inhibition (n = 2), and FGFR inhibition (n = 1), leading to 2 partial responses (PR, 7%), and 18 stable diseases (SD, 64%). These patients had a median PFS of 3.1 months (95% CI 1.8–4.3 months), which was significantly better than the 1.9 months (95% CI 1.5–2.3 months; *P* = 0.034) in patients who did not receive matched therapy, as shown in Fig. 2a.

### Survival

The median OS for these 79 patients was 8.9 months (95% CI 6.957–10.843 months) from the initial CCTT visit, and was 41.4 months (95% CI 37.231–45.569 months) from the initial metastasis diagnosis. After their initial phase I clinic visit, patients who received matched therapy showed significantly longer survival than those who did not received matched therapy (10.3 vs. 7.4 months, *P* = 0.016; Fig. 2b). Further analysis of patients treated with matched therapy showed that patients who received the matched therapy including an anti-angiogenic agent (26 months, 95% CI 0.0–55.2 months) lived significantly longer than those who did not (7.4 months, 95% CI 4.9–9.9 months; *P* = 0.04) (Fig. 2c).

**Figure 3:**
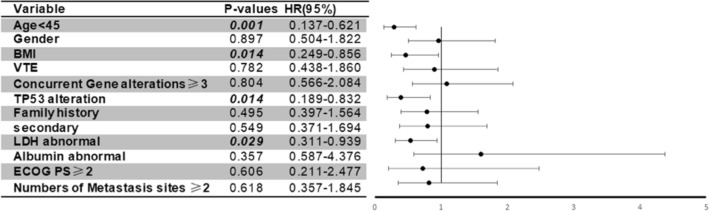
Forest plot of multivariate analysis of risk factors (age < 45 years old, BMI ≥ 25 kg/m^2^, TP53 mutation, and elevated LDH) for overall survival after initial metastasis diagnosis.

**Figure 4:**
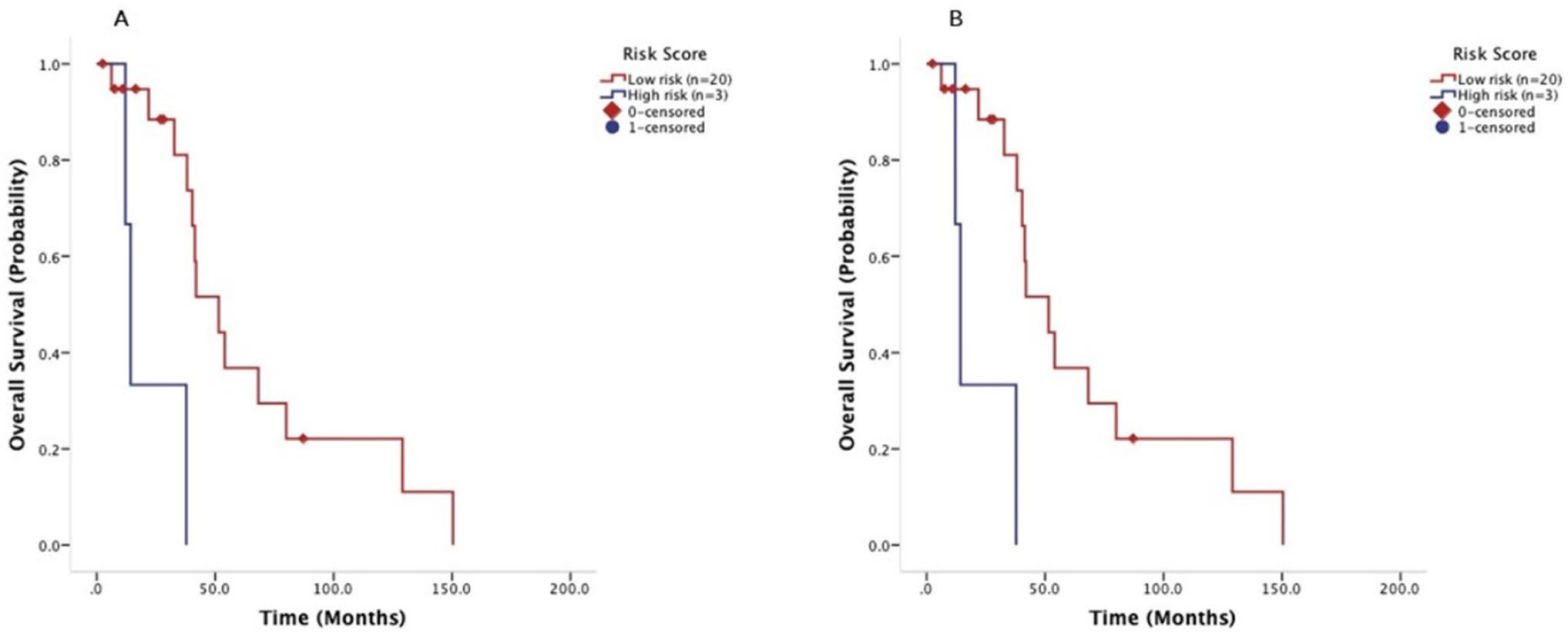
A prognostic model established factors by multivariate analysis of risk factors (age < 45 years old, BMI ≥ 25 kg/m^2^, TP53 mutation, and elevated LDH) for overall survival after initial metastasis diagnosis, was validated in 56 patients with metastatic cancer harboring CCNE1 amplification who received phase I trial therapy (**A**) and in 23 patients who did not receive phase I trial therapy (**B**) Kaplan–Meier curves estimate overall survival after initial phase I trial clinic visit, stratified by risk score (low-risk group with ≤ 1 risk factor and high-risk group with > 1 risk factors).

Univariate analyses revealed that age < 45 years old, body mass index (BMI) ≥ 25 kg/m^2^ and elevated LDH > upper limit of normal were prognostic factors for worse OS after metastasis (*P* = 0.009, *P* = 0.03 and *P* = 0.04, respectively) (Table 2). Multivariate analysis showed that age < 45 years old, BMI < 25 kg/m^2^, *TP53* mutation, and elevated LDH were significant prognostic factors for predicting shorter survival after metastases (*P* = 0.001, *P* = 0.01, *P* = 0.01 and *P* = 0.03, respectively) (Fig. 3). On the basis of the multivariate analysis results, these four parameters were then extracted by using binary subgroups (no = 0, yes = 1) to explore a risk prognostic model to predict OS after their initial CCTT visit. This model classified the patients who received phase I trial therapy into two risk cohorts: those in the low-risk group with the risk factor score ≤ 1 (n = 22) had a median OS of 55.1 months (95% CI 34.5–75.7 months) after their initial diagnosis of metastatic disease, significantly longer than the 30.2 months (95% CI 15.1–45.3 months) observed in patients in the high-risk group with the risk factor score > 1 (n = 34; *P* = 0.001) (Fig. 4a). Similarly, in the cohort of patients who were referred to a phase I clinical trial but did not receive phase I trial therapy at the CCTT, patients in the low-risk group (n = 20) had a median OS of 51.5 months (95% CI 33.6–69.4 months), which was significantly better than the 14.3 months (95% CI 10.8–17.8 months, *P* = 0.001) in those in the high-risk group (n = 3) (Fig. 4b).

**Table 2:** Univariate analysis of risk factors for overall survival.

Risk factors	Median	95% CI	*P*-value
**Age < 45 years old**			
Yes	30.2	3.9–56.5	0.009
No	41.8	35.6–48.0	
**Gender**			
Female	40.3	35.4–45.2	0.866
Male	41.4	29.2–53.6	
**Race**			
White	40.3	32.4–48.2	0.738
Black	41.7	25.8–57.6	
Asian	41.8	0.0–84.1	
Others	19.2	0.0–47.9	
**Previous platinum treatment**			
Yes	38.9	31.6–71.8	0.058
No	51.7	34.3–43.5	
**Body mass index**			
≥ 25	37.8	29.3–46.3	0.033
< 25	46.3	35.4–57.2	
**Venous thromboembolism**			
Yes	33.3	35.4–47.4	0.890
No	41.4	13.7–52.9	
**Surgery**			
Yes	44.3	38.2–49.4	0.053
No	23.7	10.8–36.6	
**Radiotherapy**			
Yes	41.7	37.0–45.5	0.865
No	41.4	33.0–50.4	
**Prior systemic therapy**			
1	27.4	7.5–47.5	0.141
≥ 2	41.9	35.7–48.1	
**Prior anti-angiogenic therapy**			
Yes	44.3	40.0–48.6	0.779
No	38.0	32.0–44.0	
**Prior immunology treatment**			
Yes	44.4	30.7–58.1	0.508
No	40.3	36.1–44.5	
**Family history of cancer**			
Yes	44.4	36.4–52.4	0.250
No	39.5	34.9–44.1	
**Second primary tumor**			
Yes	41.7	34.4–49.0	0.882
No	40.3	36.4–44.2	
**LDH > upper limit of normal**			
Yes	33.3	19.3–47.3	0.039
No	46.3	38.3–54.4	
**Albumin < lower limit of normal**			
Yes	40.3	35.9–44.7	0.541
No	58.7	32.5–84.9	
**ECOG performance status**			
≤ 1	41.4	37.3–45.5	0.760
> 1	39.5	0.0–87.2	
**Concurrent gene aberrations**			
< 3	44.3	38.7–49.9	0.786
≥ 3	38.0	26.2–49.8	
**TP53 mutation**			
Yes	38.9	33.5–44.3	0.125
No	44.8	39.2–50.4

## Discussion

In this retrospective review of cancer patients in a designated phase I clinical trial, several observations have been made. First, *CCNE1* amplification was detected in a broad spectrum of human malignancies, more frequently in high-grade malignancies, which was in consistence with its biological functions in cell proliferation and differentiation. CCNE1 amplifications have been observed in more than 7.5% of many tumor types: uterine, breast, ovarian, pancreatic, bladder, gastric, esophageal, lung cancers and sarcoma^8^. Cyclin E1 controls the transition of quiescent cells to the cell cycle, as well as epithelial-mesenchymal transitions. Overexpression of cyclin E1 induces replication stress, forcing premature entry into the S phase, and functioning as a key factor for c-myc driven tumorigenesis^25^. Activated cycle E1 further upregulates its own expression by phosphorylating Rb and releasing more E2F independent of mitogenic stimuli. This positive feedback network drives the mitotic transition from the G1 to the S phase, resulting in enhanced cellular proliferation and tumor progression^26^. This aggressive phenotype partly contributes to poor survival in patients with metastatic malignancies harboring *CCNE1* amplification, in addition to *CCNE1* amplification mediated resistance to chemotherapy^8^.

Secondly, we observed a clinical benefit in PFS and OS in patients who were treated with matched therapy targeting concurrent mutations in this cohort of patients. We do not yet have investigational agents for direct inhibition of *CCNE1* since the oscillations of the cyclins through the cell cycle are necessary for cell proliferation^27^. However, we investigated whether we would be able to target other concurrent genetic mutations or develop novel strategies to treat *CCNE1* amplified cancer cells via synthetic lethal interaction other therapeutics, such as Wee1 kinase or CDK2 kinase inhibition^28–30^. Data observed from this retrospective study did support the use of targeting concurrent genetic mutations in patients with advanced malignancies harboring undruggable genetic aberrations.

Another interesting finding we have observed in this study was that antiangiogenic therapy provided significantly better OS in patients with *CCNE1* amplified malignancies. This result might be due to frequent concurrent *TP53* mutations in these patients. Among many candidate pathways, the vascular endothelial growth factor (VEGF) pathway serves an important survival function in cancer cells with a mutated *TP53*^31,32^. *TP53* mutations in tumor cells increased the level of hypoxia-induced factor-1α (HIF-1α) and augmented HIF-1α-dependent transcriptional activation of the VEGF gene in response to hypoxia^33^. Cancer cells with mutated p53 have accelerated tumor growth associated with increased VEGF expression and neovascularization^34,35^, which serves an important survival pathway, resulting in a therapeutic advantage of VEGF-inhibition in patients with p53 mutant malignancies, which was supported by our finding that VEGF inhibition-based therapies led to significantly longer PFS in patients with a mutated *TP53* than in patients with wild-type *TP53*^36–39^.

As for the use of the therapeutic strategy of synthetic lethality, it was noted that in a phase II clinical of a Wee1 kinase inhibitor adavosertib in patients with *CCNE1* amplified advanced solid tumors (NCT03253679), 7 PRs were observed, for an overall response rate of approximately 26%. The median PFS was 4 months and 1-year OS was 55%. In 13 patients with measurable high-grade serous ovarian cancer, 5 (39%) achieved PR and 8 (62%) had SD ≥ 6 months/PR^40^. These results have encouraged us to investigate the development of novel effective therapies by combining targeted therapies against concurrent genetic mutations such as anti-angiogenesis with treatments based on synthetic lethality strategies such as Wee1 kinase inhibition. In addition, the introduction of immunotherapy to these potentially promising therapeutics targeting patients with *CCNE1* amplified malignancies warrants further consideration.

Finally, we conducted multivariate analyses that revealed four prognostic factors for poor outcomes in this cohort of patients after metastatic disease was initially diagnosed: age < 45 years old, BMI ≥ 25 kg/m^2^, presence of the *TP53* mutation, and elevated LDH. Patients in the low-risk group with the risk factor score ≤ 1 had a significantly longer median OS of 55.1 months (95% CI 34.5–75.7 months) after they were initially diagnosed to have metastatic disease than those in the high-risk group with the risk factor score > 1 who had a median OS of 30.2 months (95% CI 15.1–45.3 months). The parameters held true when we applied this model to patients who were initially seen at the phase I trials program but did not receive phase I trial therapy at the CCTT. These prognostic factors have been confirmed to be associated with poor outcome in other reports^38,39,41^.

In considering the clinical importance and relevance of our observations, several limitations should be beard in mind. First, as it is a retrospective study, the selection bias of patient referral to our phase I clinical trials program may limit the generalizability of our findings. Second, small sample sizes in the subgroup analyses may limit the validity of these statistical assessments on other prognostic factors such as the status of concurrent mutations. Finally, the actual molecular profiles were performed at various times and at different CLIA-certified molecular diagnostic laboratories and used next-generation sequencing for different sets of genes, which made it difficult to analyze status of concurrent genetic aberration. Therefore, the findings from this retrospective study should be considered preliminary evidence only for the purpose of hypothesis generation, and require further validation in larger prospective studies.

**Figure 5:**
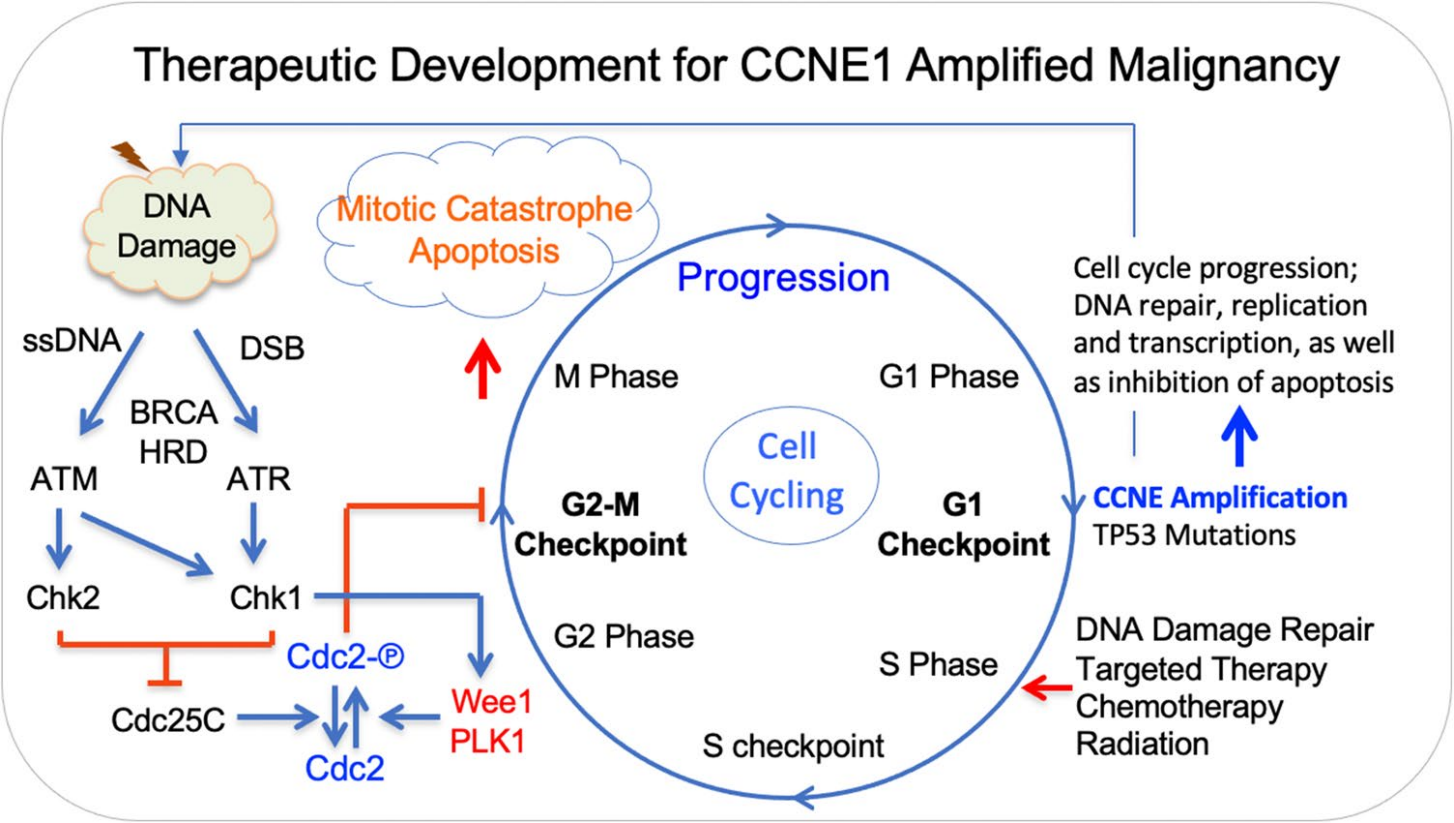
A sketch of therapeutic development for CCNE1 amplified malignancies. Cycle E overexpression due to CCNE1 amplification and concurrent mutant p53 due to TP53 mutation promote progression from the G1 phase into S phase, providing therapeutic opportunity through synthetic lethality, chemotherapy, targeted therapy and radiation to enhance mitotic catastrophe and apoptosis.

In summary, the current study, to the best of our knowledge, was the first to analyze clinical outcome in patients with various advanced *CCNE1* amplification cancers who were referred to a designated phase I trial clinic. Our study showed that *CCNE1* amplification frequently occurred in high-grade adenocarcinoma and that more than 90% of *CCNE1* amplification coexisted with other genomic aberrances. Matched therapy targeting concurrent mutations might provide clinical benefit. Multivariate analyses showed four poor prognostic factors: age < 45 years old, BMI ≥ 25 kg/m^2^, presence of *TP53* mutation, and abnormally elevated LDH, which was able to classify patients with metastatic malignancies harboring *CCNE1* amplification into either the low-risk group with ≤ 1 risk factor or the high-risk group with > 1 risk factor. Our preliminary data showed the role of concurrent genetic mutations, synthetic lethality and antiangiogenesis on patient survival, which may support future development of promising strategic therapeutics for patients with metastatic malignancies harboring *CCNE1* amplification, as outline in Fig. 5.
